# Supplementary material for: Differentiation of Lacticaseibacillus zeae Using Pan-Genome Analysis and Real-Time PCR Method Targeting a Unique Gene
Source: Foods. 2021 Sep 7;10(9):2112. doi: 10.3390/foods10092112 (PMC8468013; doi:10.3390/foods10092112)
Supplement: Supplementary file 1 [file foods-10-02112-s001.zip › foods-1333019-supplementary.pdf]

Supplementary Materials

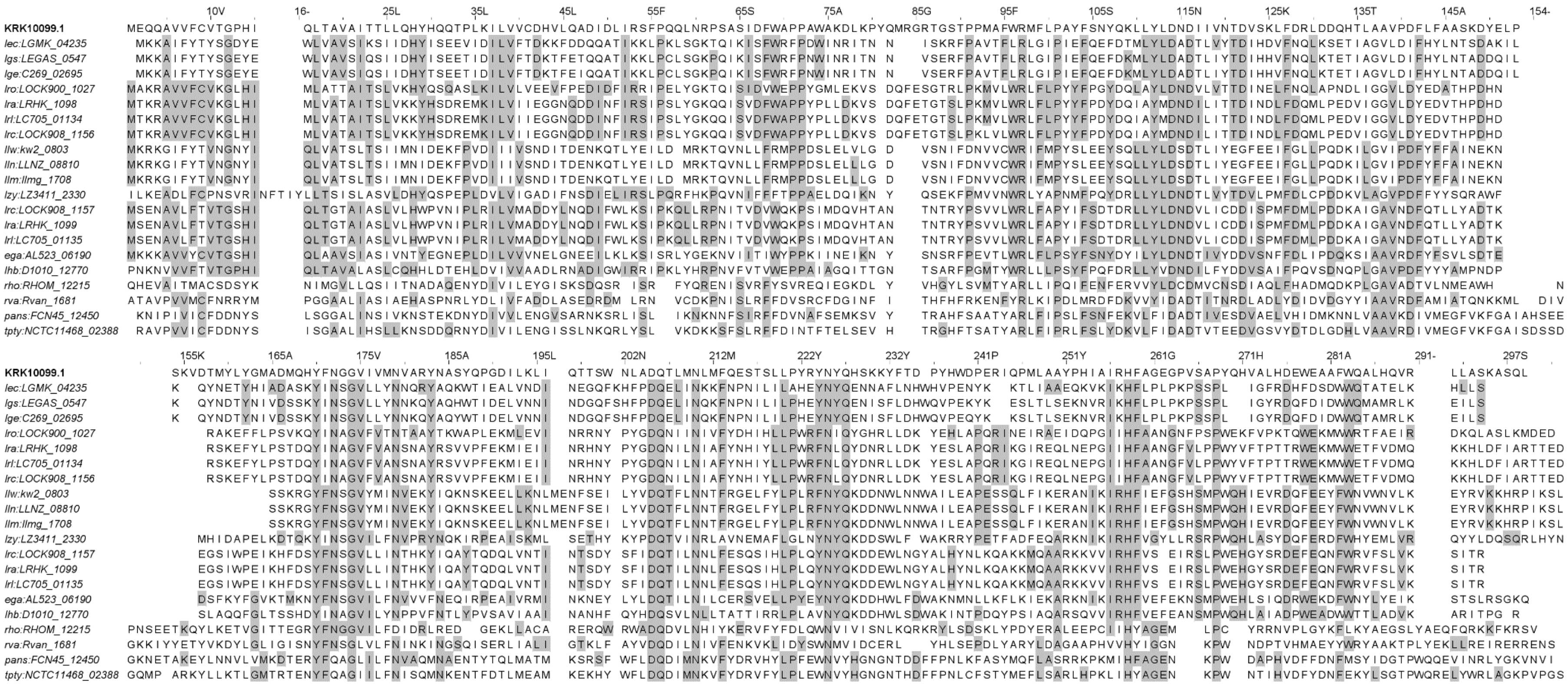

Figure S1. Sequence alignment for glycosyltransferase between *L. zeae* and other strains. The gray background indicates the sequence consistent with the glycosyltransferase family 8 (accession no. KRK10099.1) of *L. zeae*. lec:LGMK_04235, glycosyltransferase of *Leuconostoc* sp. C2; lgs:LEGAS_0547, glycosyltransferase of *Leu. gelidum* subsp. *gasicomitatum*; lge:C269_02695, glycosyltransferase of *Leu gelidum* JB7; lro:LOCK900_1027, Putative glycosyltransferase of *L. rhamnosus* LOCK900; lra:LRHK_1098, glycosyltransferase 8 family protein of *L. rhamnosus* ATCC 8530; lrl:LC705_01134, glycosyltransferase, group 8 of *L. rhamnosus* Lc 705; lrc:LOCK908_1156, Putative glycosyltransferase of *L. rhamnosus* LOCK908; llw:kw2_0803, glycosyltransferase GT8 family of *Lc. lactis* subsp. *cremoris* KW2;

lln:LLNZ_08810, putative glycosyltransferase of *Lc. lactis* subsp. *cremoris* NZ9000; llm:llmg_1708, putative glycosyltransferase of *Lc. lactis* subsp. *cremoris* MG1363; lzy:LZ3411_2330, Aspartate aminotransferase of *L. zymae*; lrc:LOCK908_1157, glycosyltransferase, family 2 of *L. rhamnosus* LOCK908; lra:LRHK_1099, glycosyltransferase 8 family protein of *L. rhamnosus* ATCC 8530; lrl:LC705_01135, glycosyltransferase, group 8 of *L. rhamnosus* Lc 705; ega:AL523_06190, glycosyltransferase family 8 of *E. gallinarum*; lhb:D1010_12770, glycosyltransferase family 8 protein of *S. harbinensis*; rho:RHOM_12215, glycosyltransferase of *Ro. hominis*; rva:Rvan_1681, glycosyltransferase family 8 of *Rh. vannielii*; pans:FCN45_12450, DUF4422 domain-containing protein of *Pantoea* sp. SO10; tpty:NCTC11468_02388, gspA; General stress protein A of *T. ptyseos*

Table S1. Similarities for glycosyltransferase between *L. zeae* and other strains

Rank	Org code	Full name	Gene name	Description	Bits	E-val	Identity
1	ega	*Enterococcus gallinarum*	AL523_06190	glycosyl transferase family 8	218	2.00E-65	36%
2	lro	*Lacticaseibacillus rhamnosus* LOCK900	LOCK900_1027	putative glycosyltransferase	188	8.00E-54	34%
3	lrc	*Lacticaseibacillus rhamnosus* LOCK908	LOCK908_1157	glycosyl transferase, family 2	183	4.00E-52	34%
4	lra	*Lacticaseibacillus rhamnosus* ATCC 8530	LRHK_1099	glycosyl transferase 8 family protein	183	4.00E-52	34%
5	lrl	*Lacticaseibacillus rhamnosus* Lc 705	LC705_01135	glycosyl transferase, group 8	183	4.00E-52	34%
6	lhb	*Schleiferilactobacillus harbinensis*	D1010_12770	glycosyltransferase family 8 protein	182	1.00E-51	35%
7	lzy	*Lactobacillus zymae*	LZ3411_2330	aspartate aminotransferase	176	7.00E-49	35%
8	lra	*Lacticaseibacillus rhamnosus* ATCC 8530	LRHK_1098	glycosyl transferase 8 family protein	171	4.00E-47	33%
9	lrl	*Lacticaseibacillus rhamnosus* Lc 705	LC705_01134	glycosyl transferase, group 8	171	4.00E-47	33%
10	lrc	*Lacticaseibacillus rhamnosus* LOCK908	LOCK908_1156	putative glycosyltransferase	169	1.00E-46	33%
11	llw	*Lactococcus lactis* subsp. *cremoris* KW2	kw2_0803	glycosyl transferase GT8 family	118	5.00E-27	26%
12	lln	*Lactococcus lactis* subsp. *cremoris* NZ9000	LLNZ_08810	putative glycosyltransferase	118	5.00E-27	26%
13	llm	*Lactococcus lactis* subsp. *cremoris* MG1363	llmg_1708	putative glycosyltransferase	118	5.00E-27	26%
14	lgs	*Leuconostoc gelidum* subsp. *gasicomitatum*	LEGAS_0547	glycosyl transferase	112	1.00E-25	26%
15	lge	*Leuconostoc gelidum* JB7	C269_02695	glycosyl transferase	112	2.00E-25	26%
16	lec	*Leuconostoc* sp. C2	LGMK_04235	glycosyl transferase	110	1.00E-24	26%
17	rva	*Rhodomicrobium vannielii*	Rvan_1681	glycosyl transferase family 8	86.7	4.00E-16	23%
18	pans	*Pantoea* sp. SO10	FCN45_12450	DUF4422 domain-containing protein	83.2	1.00E-14	25%
19	tpty	*Tatumella ptyseos*	NCTC11468_02388	gspA; General stress protein A	83.2	1.00E-14	24%
20	rho	*Roseburia hominis*	RHOM_12215	glycosyl transferase	77.4	3.00E-13	24%
